# Chemical switching of low-loss phonon polaritons in α-MoO_3_ by hydrogen intercalation

**DOI:** 10.1038/s41467-020-16459-3

**Published:** 2020-05-27

**Authors:** Yingjie Wu, Qingdong Ou, Yuefeng Yin, Yun Li, Weiliang Ma, Wenzhi Yu, Guanyu Liu, Xiaoqiang Cui, Xiaozhi Bao, Jiahua Duan, Gonzalo Álvarez-Pérez, Zhigao Dai, Babar Shabbir, Nikhil Medhekar, Xiangping Li, Chang-Ming Li, Pablo Alonso-González, Qiaoliang Bao

**Affiliations:** 1 Department of Materials Science and Engineering, and ARC Centre of Excellence in Future Low-Energy Electronics Technologies (FLEET), Monash University, Clayton, Australia; 2 State Key Laboratory of Functional Materials for Informatics, Shanghai Institute of Microsystem and Information Technology, Chinese Academy of Sciences, Shanghai, China; 3 Guangdong Provincial Key Laboratory of Optical Fiber Sensing and Communications, Institute of Photonics Technology, Jinan University, Guangzhou, China; 4 Laboratory of Automobile Materials of MOE, School of Materials Science and Engineering, Jilin University, Changchun, China; 5 Joint Key Laboratory of the Ministry of Education, Institute of Applied Physics and Materials Engineering (IAPME), University of Macau, Macau SAR, China; 6 Departamento de Física, Universidad de Oviedo, Oviedo, Spain; 7 Center of Research on Nanomaterials and Nanotechnology, CINN (CSIC-Universidad de Oviedo), El Entrego, Spain; 8 Institute of Advanced Cross-field Science, College of Life Science, Qingdao University, Qingdao, China

**Keywords:** Two-dimensional materials, Nanophotonics and plasmonics

## Abstract

Phonon polaritons (PhPs) have attracted significant interest in the nano-optics communities because of their nanoscale confinement and long lifetimes. Although PhP modification by changing the local dielectric environment has been reported, controlled manipulation of PhPs by direct modification of the polaritonic material itself has remained elusive. Here, chemical switching of PhPs in α-MoO_3_ is achieved by engineering the α-MoO_3_ crystal through hydrogen intercalation. The intercalation process is non-volatile and recoverable, allowing reversible switching of PhPs while maintaining the long lifetimes. Precise control of the intercalation parameters enables analysis of the intermediate states, in which the needle-like hydrogenated nanostructures functioning as in-plane antennas effectively reflect and launch PhPs and form well-aligned cavities. We further achieve spatially controlled switching of PhPs in selective regions, leading to in-plane heterostructures with various geometries. The intercalation strategy introduced here opens a relatively non-destructive avenue connecting infrared nanophotonics, reconfigurable flat metasurfaces and van der Waals crystals.

## Abstract (web summary)

Phonon polaritons hold promises for nanophotonic applications but external control of phonon polaritons remains challenging. Here, the authors achieve reversible and non-volatile switching of phonon polariton by modifying crystal structure and lattice vibrations via hydrogenation.

## Introduction

Polaritons are hybrid electromagnetic waves originating from the coupling of light and dipole-like excitations in matter and include phonon polaritons (PhPs), plasmon polaritons, and exciton polaritons^1–4^. Among these polaritonic modes, PhPs with strong field confinement, low loss, and a long lifetime can enable a wide range of applications in light transmission, electro-optical modulation, sub-diffraction focusing, and imaging, and molecular detection^5–8^. However, to best utilize PhPs, external control of their properties is needed, which is a difficult task owing to the wide bandgap (~2–10 eV) in polar crystals, making the common electrical-gating method impractical.

Although a few attempts have been made^9,10^, effective tuning of PhPs remains challenging. Recently, PhP manipulation has been investigated by controlling the dielectric environment through the fabrication of vertical heterostructures of van der Waals (vdW) crystals (hexagonal boron nitride (h-BN)/black phosphorous^11^ and MoS_2_/SiC^12^), polaritonic metasurfaces in combination with phase change materials (h-BN/VO_2_^13–15^, germanium antimony telluride (GeSbTe)/quartz^16^ and h-BN/GeSbTe^17^), or polar materials suspended in air^18^. The latter enables improvement of the PhP figures of merit but imposes limitations on the device architecture and functionality.

Very recently, in-plane anisotropic and ultra-low-loss PhPs in the natural vdW semiconductor crystal α-phase MoO_3_ (α-MoO_3_) have been reported^19,20^. As a typical transition metal oxide, α-MoO_3_ possesses variable chemical and physical properties and is widely studied for use in catalysis, energy storage devices, and optoelectronic devices^21–23^. In particular, the high chemical compatibility of α-MoO_3_ enables direct tuning of its optical properties by metal-atom intercalation^24^. Intercalation has been previously explored in nanometer-thick MoO_3_ slabs^25^, where metallic Sn atoms were introduced into the crystal-lattice interlayers, effectively modifying the surface propagation of PhPs. Nevertheless, realizing intercalation tuning PhPs in a controlled and reversible way is still challenging due to the large volumes of the intercalators and the solvent-based character of the process.

Here, we successfully demonstrate chemical switching of PhPs in nanometer-thick α-MoO_3_ by precisely modifying its crystal structure via hydrogen intercalation (so-called hydrogenation or protonation). Unlike changing external dielectric constants, our hydrogenation approach directly acts on the polaritonic material itself. The crystal lattice of MoO_3_ is rearranged owing to the formation of type I H_*x*_MoO_3_ (0.2 < *x* < 0.4), giving rise to perturbed phonon oscillations and the disappearance of PhP propagation. Importantly, following a deintercalation (also known as dehydrogenation or deprotonation) process, the crystal structure can fully recover to its original state without significant damage, enabling quantitatively reversible switching of low-loss PhPs. By precisely controlling the intercalation time, in-plane nanostructures that can reflect and launch PhPs are obtained in intermediate states. Furthermore, by combining our intercalation method with photolithography, we realize spatially controlled switching of PhPs. Our findings provide a chemical strategy for effective control of PhPs to develop reconfigurable polaritonic devices or topological photonic structures on a large scale.

## Results

### Structural analysis during reversible hydrogenation

**Fig. 1:**
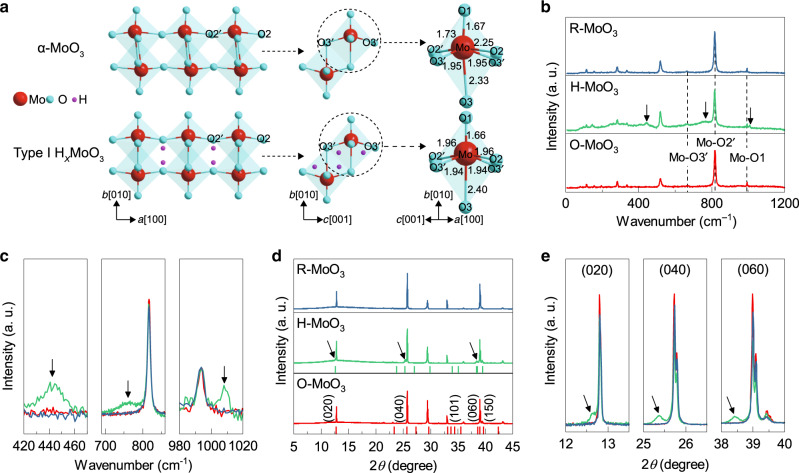
Structural characterizations during the hydrogenation/dehydrogenation process. **a** Schematic illustrations of the crystal structures (linked double layers) and bond lengths (Å, ref. ^28^) of α-MoO_3_ and type I H_x_MoO_3_. O1, O2 and O3 represent terminal oxygens, doubly coordinated bridging oxygens and triply coordinated oxygens, respectively. **b** Raman spectra of the original (O-MoO_3_), hydrogenated (H-MoO_3_), and recovered (R-MoO_3_) α-MoO_3_. Black arrows indicate the vibration peaks that emerged after hydrogenation. **c** Magnified Raman spectra (normalized) of O-MoO_3_, H-MoO_3_, and R-MoO_3_ in **b**. **d** XRD pattern of the original, hydrogenated and recovered α-MoO_3_. Red and green tick marks represent the peaks in ICDD PDF card no. 05–0508 and 70–0615, respectively. Black arrows indicate the diffraction peaks originating from type I H_x_MoO_3_. **e** Comparison of the (020), (040), and (060) diffraction peaks (normalized) in **d**.

As a typical vdW material, α-MoO_3_ is composed of double layers of linked and distorted MoO_6_ octahedra^26^, as shown in Fig. 1a. The nanometer-thick α-MoO_3_ (original α-MoO_3_, hereafter termed O-MoO_3_) flakes studied in this work were synthesized by a chemical vapor deposition (CVD) method, followed by a mechanical exfoliation process (see Methods and Supplementary Fig. 1, in which different characterizations of the as-prepared α-MoO_3_ flakes confirm their orthorhombic crystal structure). The hydrogen intercalation process was conducted by treating the flakes with a hydrogen plasma. To avoid irreversible damage to the crystal, the exposure time was controlled at 10 s (see Methods and Supplementary Fig. 2). Four typical hydrogen molybdenum bronzes (H_*x*_MoO_3_, 0 < *x* ≤ 2) with different compositions and structures can be obtained after α-MoO_3_ hydrogenation^27^. In our experiment, we obtained type I H_*x*_MoO_3_ (orthorhombic, 0.2 < x < 0.4) in hydrogenated α-MoO_3_ (hereafter, termed H-MoO_3_), as revealed by Raman and X-ray diffraction (XRD) analysis. In the MoO_6_ octahedron, the oxygen atoms can be divided into three types according to their site (Fig. 1a). As shown in Fig. 1b, c, the Raman shifts at 667, 818, and 994 cm^−1^ are assigned to the stretching vibration modes of the Mo–O3’, Mo–O2’, and Mo–O1 bonds, respectively. After hydrogenation, owing to a structural rearrangement, the peaks at 818 and 994 cm^−1^ shift to 760 (broadband) and 1008 cm^−1^ (black arrows in Fig. 1b), respectively, agreeing well with the Raman spectrum of type I H_x_MoO_3_^28^. Another stretching vibration peak of the Mo–O3 bond at 441 cm^−1^, which is hardly observable in O-MoO_3_, emerges after hydrogenation. Given that the Raman peaks at 667, 818, and 994 cm^−1^ remain discernible in H-MoO_3_, even those with weaker intensities (Supplementary Fig. 3), the hydrogenated product is a mixture of type I H_x_MoO_3_ and α-MoO_3_. In addition, the XRD spectra of O-MoO_3_ (Fig. 1d,e) correspond to the *Pbnm* space group of orthorhombic α-MoO_3_ (ICDD PDF: 05–0508) with lattice constants *a* = 3.97 Å, *b* = 13.86 Å, and *c* = 3.71 Å. After hydrogenation, new peaks arise at 12.6°, 25.3°, and 38.3° (black arrows), which are attributed to the (020), (040), and (060) planes of orthorhombic H_0.31_MoO_3_ (ICDD PDF: 70–0615). This result unambiguously proves the formation of type I H_x_MoO_3_ with calculated lattice constants *a* = 3.93 Å, *b* = 14.02 Å, and *c* = 3.74 Å.

The as-prepared H_x_MoO_3_ is stable at room temperature and can last for several months in the ambient atmosphere (Supplementary Fig. 4). However, we can apply a simple thermal treatment (see Methods) to induce deintercalation, which proceeds as

1 $${\mathrm{H}}_{{x}}{\mathrm{MoO}}_3 + {\mathrm{O}}_2 \to {\mathrm{MoO}}_3 + {\mathrm{H}}_2{\mathrm{O}}$$

Both the Raman and XRD spectra of the recovered α-MoO_3_ (hereafter, termed R-MoO_3_) agree excellently with those of O-MoO_3_, demonstrating that the H-MoO_3_ crystal structure is reconfigured to α-MoO_3_ after dehydrogenation. Moreover, X-ray photoelectron spectroscopy (XPS) characterizations (Supplementary Fig. 5 and Supplementary Table 1) provide solid evidence for the reversibility of the chemical composition changes that occur during the intercalation/deintercalation process. Compared with the metal-atom intercalation, our approach thus affords an effective and relatively non-destructive strategy to reversibly modify the composition and structure of nanometer-thick transition metal oxide flakes.

### Reversible switching of PhPs

**Fig. 2:**
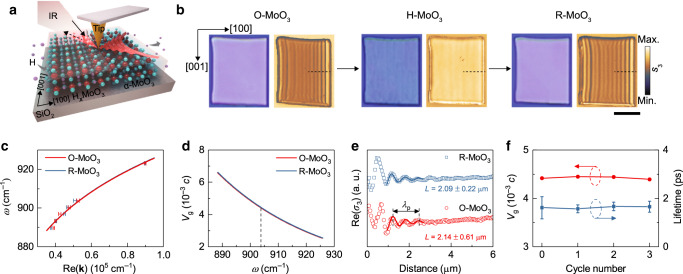
Reversible switching of PhPs in the L-RB of α-MoO_3_. **a** Schematic of the s-SNOM measurement and PhP propagation in a typical H-MoO_3_/α-MoO_3_ in-plane heterostructure. **b** Optical micrographs and s-SNOM amplitude images (890 cm^−1^) of a MoO_3_ flake on a SiO_2_/Si substrate before intercalation, after intercalation (10 s) and after deintercalation. Scale bar = 5 μm. **c** PhP dispersion data and fitting results (solid lines) along the [100] direction. The error bars define the 95% confidence intervals. **d** Group velocities of PhPs in original and recovered flakes extracted from the fitting results in **c**. **e** Extracted real-part near-field line traces and fitting curves (solid lines) at 903 cm^−1^. **f** Comparison of the group velocities and lifetimes of PhPs (903 cm^−1^) after three reversible intercalation cycles. The error bars of lifetimes were calculated based on the errors of propagation lengths originating from the standard deviation of fitting.

The structural reversibility during hydrogenation/dehydrogenation processes enables direct manipulation of the vibration-dependent PhPs in nanometer-thick α-MoO_3_ slabs. To study the polaritonic response of our samples, we used scattering-type scanning near-field optical microscopy (s-SNOM, Fig. 2a), which enables direct mapping of the near field in terms of the amplitude and phase and the sample topography by illuminating the sample with mid-infrared light at different frequencies. First, we measured the samples in the lower Reststrahlen band (L-RB, 818–974 cm^−1^) of α-MoO_3_, where PhPs are known to propagate with a hyperbolic wavefront along the [100] direction^19,20,25^. Figure 2b shows s-SNOM amplitude images (third-harmonic signals, *s*_3_) at frequency *ω* = 890 cm^−1^ together with optical micrographs of O-MoO_3_, H-MoO_3_, and R-MoO_3_ flakes with the thicknesses of 220 nm. As expected, for O-MoO_3_, we observe interference fringes in the s-SNOM image, revealing the excitation of PhPs propagating parallel to the [001] direction with a polariton wavelength *λ*_p_ = 1.61 ± 0.01 μm, which well matches previous work^19^. After intercalation with hydrogen (H-MoO_3_), we observe a weaker contrast in the optical image, suggesting a varied optical behavior. More importantly, no regular interference fringes can be observed in the s-SNOM image (Supplementary Fig. 6), implying that PhPs are not excited. After dehydrogenation, the initial contrast of the flake in the optical image is recovered, and parallel fringes in the s-SNOM image are again observed, revealing the excitation of PhPs.

For a quantitative analysis of the PhP behavior, we compared the group velocities (*v*_g_), propagation lengths (*L*), and lifetimes (*τ*) of PhPs in the original and recovered α-MoO_3_ flakes. First, we extracted the PhP dispersions (*ω*(Re(**k**)), where **k** is the complex-valued wavevector) along the [100] direction (dashed lines in Fig. 2b) at different frequencies by fitting the line scans obtained from s-SNOM amplitude and phase images using the function^29^:

2 $$\sigma \left( x \right) = A\frac{{{\mathrm{e}}^{i2{\mathbf{k}}x}}}{{\sqrt x }} + B\frac{{{\mathrm{e}}^{i{\mathbf{k}}x}}}{{x^d}}$$

where *σ* is the complex-valued optical signal, *d* (~1) is variable decay, *A* and *B* are the parameters for tip- and edge-launched PhPs, respectively. We used the function *y* = *ax*^*b*^ to fit the dispersion data (solid lines in Fig. 2c, see Supplementary Table 2) and then performed a numerical derivation process on the fitting curve, as the group velocity can be extracted by *v*_g_ = ∂*ω/*∂Re(**k**)^19^. The resulting group velocities of PhPs in the original and recovered α-MoO_3_ flakes are shown in Fig. 2d. We observe similar values of 4.42 × 10^−3^ *c* and 4.45 × 10^−3^ *c* at *ω* = 903 cm^−1^ for the original and recovered flakes, respectively, where *c* is the speed of light in vacuum. Then we calculate the polariton lifetime by *τ* = *L*/*v*_g_ (see Supplementary Note 1 for more details). Accordingly, the line profiles at *ω* = 903 cm^−1^ and the fitting results are plotted in Fig. 2e, where the propagation lengths of PhPs in the original and recovered α-MoO_3_ are 2.14 ± 0.61 μm and 2.09 ± 0.22 μm, respectively. The calculated lifetime of PhPs in O-MoO_3_ (1.61 ± 0.46 ps) is comparable to that in R-MoO_3_ (1.57 ± 0.16 ps), suggesting high-fidelity recovery of hyperbolic PhPs after dehydrogenation. The PhP behavior remains almost unchanged even after three intercalation/deintercalation cycles, as shown in Fig. 2f (Supplementary Fig. 7).

**Fig. 3:**
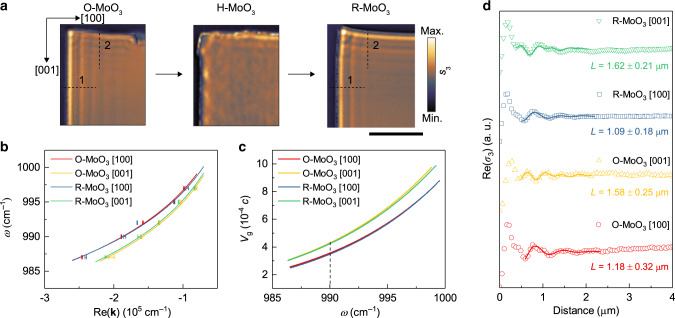
Reversible switching of PhPs in the U-RB of α-MoO_3_. **a** Optical micrographs and s-SNOM amplitude images (990 cm^−1^) of original, intercalated, and recovered MoO_3_ flakes. Scale bar = 5 μm. **b** PhP dispersion data and fitting results (solid lines) along both [100] and [001] directions (dashed lines in **a**). The error bars represent the 95% confidence intervals. **c** Group velocities of PhPs extracted from the fitting results in **b**. **d** Comparison and fitting (solid lines) of extracted line traces.

In addition to PhPs in the L-RB, PhPs with elliptical propagation in the upper Reststrahlen band (U-RB, 960–1010 cm^−1^) of α-MoO_3_ can also be reversibly switched through hydrogenation while maintaining their extraordinary properties (strong field confinement and ps lifetimes^19^). As shown in Fig. 3a, PhPs propagate along both the [100] and [001] directions in O-MoO_3_ and R-MoO_3_ owing to the elliptical PhP propagation. We compared the performances of PhPs in the original and recovered α-MoO_3_ flakes (140 nm) along the two directions (Fig. 3b–d). In the [100] direction (dashed line 1 in Fig. 3a), the PhP group velocities and lifetimes are 0.36 × 10^−3^ *c* and 10.93 ± 2.96 ps (original) and 0.35 × 10^−3^ *c* and 10.38 ± 1.71 ps (recovered) at 990 cm^−1^, respectively. Comparable characteristics are also found in the [001] direction (dashed line 2), where the group velocities (lifetimes) in the original and recovered slabs are 0.43 × 10^−3^ *c* (12.25 ± 1.94 ps) and 0.42 × 10^−3^ *c* (12.86 ± 1.67 ps), respectively. These results demonstrate that the hydrogen intercalation/deintercalation method enables robust, permanent (several months) and reversible switching of PhPs in α-MoO_3_ both in the L- and U-RB.

### Analysis of intermediate states during hydrogenation

**Fig. 4:**
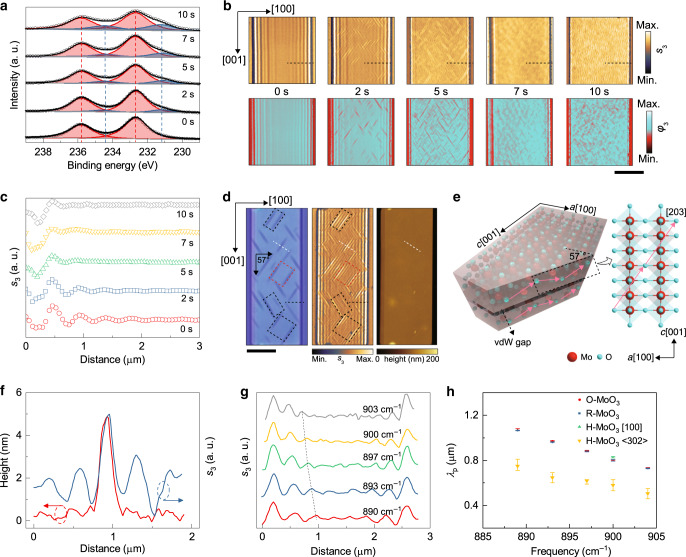
Analysis of the intercalation intermediates. **a** XPS data and proportions of Mo^6+^ (red) and Mo^5+^ (blue) in the hydrogenated intermediates. **b** Normalized amplitude images (top, 900 cm^−1^) and corresponding phase images (bottom) of a MoO_3_ slab during hydrogenation. Dashed lines indicate the line scan direction. Scale bar = 5 μm. **c** Line scans extracted from amplitude images. **d** Optical (left), amplitude (middle, 890 cm^−1^), and height (right) images of an intermediate H-MoO_3_ slab (135 nm) prepared by a two-step hydrogen intercalation process (2+2 s). Scale bar = 5 μm. **e** Schematic illustrations of three-dimensional hydrogen diffusion pathways in α-MoO_3_. Pink arrows in the cross section (left) along the [203] direction represent the alternate zig-zag diffusion pathways in the MoO_3_ intralayers. The top view (right) indicates the diffusion pathways between the top (blue) and bottom (gray) sublayers in the (010) plane. **f** Thickness (levelled to the MoO_3_ top surface) and amplitude analysis of a single H_*x*_MoO_3_ nanostructure, taken along the white dashed line in **d**. **g** Line traces extracted from the red rectangular area in the amplitude image along the [302] direction. **h** Comparison of polariton wavelengths of PhPs in O-MoO_3_, R-MoO_3_, and H-MoO_3_ along the [100] (black dashed line) and <302> directions. The average values were obtained by averaging for four typical line scans; the error bars were calculated by subtracting average values from max or min values.

To study the hydrogen intercalation process and the evolution of the PhPs excited in nanometer-thick α-MoO_3_, we prepared H-MoO_3_ flakes with various hydrogen proportions by precisely controlling the intercalation time (i.e., hydrogen plasma treatment time, Supplementary Fig. 8). The XPS results (Fig. 4a and Supplementary Table 3) show a slowly increasing proportion of Mo^5+^ (H_*x*_MoO_3_) with prolonged intercalation time. After intercalation, we used s-SNOM to investigate the effect on the PhP properties in these samples. Figure 4b shows normalized s-SNOM amplitude (top) and phase (bottom) images of a 120 nm-thick α-MoO_3_ flake before (O-MoO_3_ (0 s)) and after 2-, 5-, 7-, and 10-s hydrogen intercalations (H-MoO_3_). The parallel interference fringes in both the amplitude and phase images reveal that the excitation of PhPs gradually vanishes with increasing intercalation time, which can be further verified by the extracted amplitude line traces (Fig. 4c) and fast Fourier transform (FFT) results (Supplementary Fig. 9). The quantitative comparison indicates that upon gradual hydrogenation, the PhP dispersion has no obvious difference, while the propagation length and lifetime gradually decrease. By comparing the Raman spectra in hydrogenated intermediate states, we attribute this chemical switching of PhPs to the disappearance of optical phonons (L-RB and U-RB) of the polar material during hydrogenation (Supplementary Note 2 and Supplementary Fig. 10).

Interestingly, we found regular acicular nanostructures in intermediate H-MoO_3_ (2 and 5 s) compounds. For a better analysis, we prepared an intercalated flake (2+2 s) by a step-by-step method (Supplementary Fig. 11). We observe needle-like nanostructures (Fig. 4d) with a weaker optical contrast extending along ~57 ± 2 ° with respect to the [100] direction (on the (010) surface), which can be assigned to the <203> direction^30^. Similar nanostructures are also observed in nanometer-thick α-MoO_3_ discs (Supplementary Fig. 12), indicating that the hydrogenation direction determined by the intrinsic crystal structure is the main reason for their nucleation orientation instead of the specific geometry of the flake. Indeed, by performing Raman intensity mapping, we discover that these nanostructures consist of type I H_*x*_MoO_3_ (Supplementary Fig. 12).

To better understand the nucleation of these nanostructures, we studied the diffusion of hydrogen atoms in type I H_*x*_MoO_3_ by performing calculations using density functional theory (DFT) (see Methods and Supplementary Note 3). The O2 sites (with an adsorption energy of 3.58 eV) are the most energetically favorable for hydrogen adsorption compared with the O1 (3.06 eV) and O3 (1.13 eV) sites (Supplementary Table 4). In addition, hydrogens are alternately located along the zig-zag chains of intralayer oxygen atoms ([001] direction) within MoO_6_ octahedra^31^. By comparing the calculated thermodynamic stabilities for hydrogen diffusion along the [203] direction and along the [001] direction, we find the former to be slightly more energetically favorable (15 meV), possibly because of the cationic repulsion (Supplementary Fig. 13). We can thus conclude that the three-dimensional hydrogen diffusion in H_x_MoO_3_ at low hydrogen concentration occurs along the [203] direction, as schematically shown in Fig. 4e, giving rise to the observed needle-like nanostructures oriented along 57 ± 2 ° with respect to the [100] direction.

Further characterization by s-SNOM of these nanostructures shows that while in terms of the topography (Fig. 4d and Supplementary Fig. 14), they are relatively hard to identify (probably because of their atomic expansion of ~4 nm caused by the rearrangement of MoO_6_ octahedra, which can be recovered after deintercalation), in the amplitude image, they show a strong near-field contrast compatible with a high degree of metallicity because of the greatly enhanced conductivity of H-MoO_3_ (Supplementary Fig. 15). Clear parallel interference fringes revealing excitation and/or reflection of PhPs are observed on both sides of the nanostructure (Fig. 4f). In fact, by extracting amplitude line scans perpendicular to the [203] direction (i.e., [302]) between two parallel nanostructures (Fig. 4g) in the red rectangular region in Fig. 4d, we obtain resonator-like oscillations^32^ with frequency-dependent wavelengths similar to PhPs along that direction. We also compare *λ*_p_ at different frequencies in O-MoO_3_, R-MoO_3_, and four typical regions in H-MoO_3_ (rectangles in Fig. 4d). As displayed in Fig. 4h, the *λ*_p_ of PhPs along the [100] direction remains similar in O-MoO_3_, R-MoO_3_, and H-MoO_3_. However, PhPs along the <302> direction in H-MoO_3_ exhibit decreased polariton wavelengths, which are much shorter than those of PhPs along the same direction in O-MoO_3_. The FFT results along the [302] direction in Supplementary Fig. 16 show two prominent peaks, which is attributed to PhPs launched by both the tip and acicular nanostructures^19^; thus, we can conclude that these hydrogenated nanostructures act as efficient reflectors and launchers of PhPs (see Supplementary Fig. 17 for U-RB) while forming part of the crystal (as defined chemically and not physically), which can be highly beneficial for the development of planar devices in nanophotonic technologies (Supplementary Note 4).

### Spatially controllable switching of PhPs

**Fig. 5:**
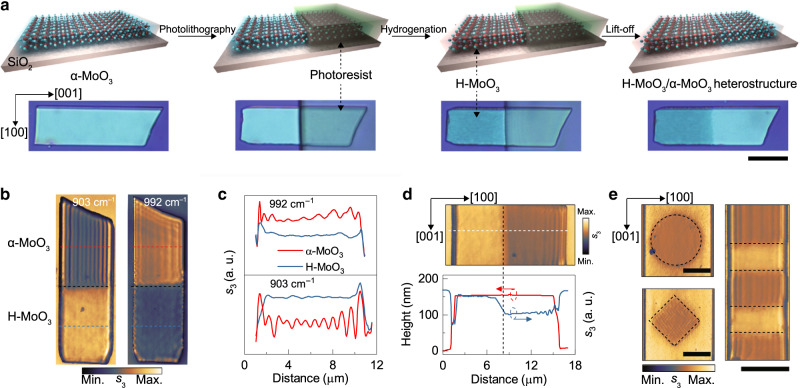
Spatially controllable switching of PhPs by selective hydrogenation. **a** Illustrations of the spatially selective hydrogen intercalation process, and corresponding microphotographs of a typical MoO_3_ flake. Scale bar = 10 μm. **b** Amplitude images of an H-MoO_3_/α-MoO_3_ in-plane heterostructure (along the [001] direction) at 903 and 992 cm^−1^. The black dashed line indicates the hydrogenation boundary. **c** Line traces extracted from amplitude images (red and blue dashed lines) in **b**. **d** Amplitude image (893 cm^−1^) and corresponding amplitude and height line scans (white dashed line) of an in-plane heterostructure (along the [100] direction). **e** Amplitude images of heterostructures with various geometries. Scale bars = 5 μm.

Finally, we studied the scalability and feasibility of spatially selective hydrogenation within α-MoO_3_ flakes. As illustrated in Fig. 5a, we combined the hydrogenation method with a conventional photolithography technique. We used direct photolithography to cover a 225 nm-thick α-MoO_3_ flakes by a photoresist with designed patterns (see Methods and Supplementary Fig. 18 and 19 for details). In this way, upon hydrogenation, only open regions of α-MoO_3_ flakes are exposed, and thus, H-MoO_3_/α-MoO_3_ in-plane heterostructures with various geometries can be achieved. Figure 5b, c shows a flake where the bottom half region has been hydrogenated and thus PhPs are switched off in both the L-RB and U-RB. In contrast, in the hydrogen-free region (α-MoO_3_), parallel interference fringes indicating PhPs are observed.

Owing to its in-plane anisotropy, α-MoO_3_-based in-plane nanostructures with different orientations are attractive for nano-optics studies and can be easily prepared using our method by designing the masked region as shown in Fig. 5d, where the boundary of the heterostructure is perpendicular to the PhP propagation direction. Furthermore, the thickness of the hydrogenated regions shows no obvious difference from that of the unhydrogenated regions, whereas the optical signal is very different, as confirmed by the extracted line traces (Fig. 5d). Figure 5e shows the potential application of our chemical switching method. By using customized patterns in photolithography, discs, squares, and more complicated arrays with various PhP dispersions can be constructed. Notably, we observe a clear reflection of PhPs at the sharp boundaries along the <203> direction (Fig. 4d and square in Fig. 5e). However, no significant reflection occurs at the boundaries along other directions (Fig. 5d and disc in Fig. 5e) because these boundaries are composed of much smaller <203>-oriented H_x_MoO_3_ nanostructures, at which PhPs are irregularly scattered, leading to unobservable reflection fringes.

## Discussion

We have demonstrated an effective chemical approach to manipulate PhPs in α-MoO_3_ by the hydrogen intercalation-induced perturbation of lattice vibrations. Reversible switching of PhPs within the α-MoO_3_ flake is achieved. The figures of merits for recovered PhPs after multiple intercalation/deintercalation cycles remain quantitatively comparable to those for the original PhPs. By precisely controlling the intercalation time, we reveal the relationship between PhPs and the crystal structures of the polar material, and in-plane nano-antennas that can reflect and launch PhPs are obtained. We also achieve spatially controllable intercalation and selective switching of PhPs by combining our intercalation process with photolithography (or possibly a shadow mask), through which in-plane heterostructures are constructed, which is difficult to realize in other PhP tuning methods (Supplementary Note 5 and Supplementary Table 5).

Our methodology establishes a proof of concept for chemically manipulating polaritons, offering opportunities for the growing nanophotonics community. We envision that in operando switching of PhPs could be realized during s-SNOM nanoimaging via tip-induced hydrogen intercalation (the so-called spillover technique^33^). The plasmonic resonance^34^, newly observed ferromagnetic properties^35^, and high conductivity of hydrogen-doped MoO_3_ enable analysis of the interaction of PhPs with plasmon and magnetic fields. Furthermore, using other intercalators, such as ions (Li^+^, Na^+^)^36,37^, metal atoms (Co, Fe)^38,39^, and polymers^40^, programmable in-plane heterostructures and flat metasurfaces could be constructed, and applications combining scalable planar optics with photomagnetics, photoelectronics and topological photonics could be realized.

## Methods

### Preparation of α-MoO_3_ flakes

α-MoO_3_ bulk crystals were grown by a CVD method. In brief, 0.1 g of MoO_3_ particle (Alfa Aesar) was placed in the middle of a quartz tube (2 inches in diameter), where the temperature was 780 °C. Ar (200 standard cubic centimetres per minute (sccm)) and O_2_ (50 sccm) were used as carrier gases to reduce the oxygen vacancies. α-MoO_3_ bulk crystals were collected at the downstream position where the temperature was ~550 °C. Nanometer-thick α-MoO_3_ flakes were mechanically exfoliated and then transferred to a SiO_2_ (300 nm)/Si substrate.

### Reversible hydrogen intercalation/deintercalation processes

The hydrogen intercalation process was conducted by a plasma-enhanced CVD method. Typically, α-MoO_3_ flakes on a SiO_2_/Si substrate (1 × 1 cm^2^) were placed in the middle of a quartz tube (four inches in diameter), where the temperature was 150 °C. A radio-frequency plasma generator (17 mW, 13.56 MHz, VERG-500) was placed upstream of the sample at a distance of ~ 50 cm. H_2_ (5 sccm) was used as the gas source under a pressure of 15 mTorr. By controlling the reaction time, hydrogen-intercalated flakes with various hydrogen levels were obtained. The deintercalation experiment was carried out by a thermal treatment process at 350 °C for 120 min. Ar (200 sccm) and O_2_ (50 sccm) were used as carrier gases at atmospheric pressure to reconfigure the crystal structure and reduce oxygen defects. Notably, to obtain H_x_MoO_3_ flakes with different hydrogen distributions, two intercalation strategies were carried out: continuous intercalation and step-by-step intercalation.

### Spatially controllable hydrogen intercalation process

Spatially controlled hydrogenation in nanometer-thick α-MoO_3_ flakes was realized by combining intercalation with photolithography. First, a layer of photoresist (~1 μm, AZ 1512, AZ Electronic Materials) was coated on top of the surfaces of α-MoO_3_ flakes and then patterned by direct-write lithography (SF100 XPRESS). After the development process, the unexposed regions of α-MoO_3_ flakes were covered and protected by the photoresist. Then, a similar hydrogenation process was conducted, during which hydrogen was inserted into the exposed regions of flakes. Following a lift-off process, selectively intercalated flakes (i.e., in-plane H-MoO_3_/α-MoO_3_ heterostructures) with designed geometries were obtained.

### Theoretical calculations

We investigated the adsorption of hydrogen on α-MoO_3_ using DFT calculations as implemented in the Vienna ab initio simulation package. The Perdew-Burke-Ernzerhof form of the generalized gradient approximation was used to describe electron exchange and correlation. The plane-wave kinetic energy cutoff was set to 600 eV. A semiempirical function developed by Grimme (DFT-D3) was employed to describe dispersion forces^41^. All structures were relaxed until the ionic forces were < 0.01 eVÅ^−1^. For accurate calculations of the electronic structures, we used 9×3×9 Γ-centered grids for sampling the Brillouin zone to calculate the preferred adsorption site of one hydrogen on α-MoO_3_ and 5 × 5 × 3 Γ-centered grids to simulate the hydrogen diffusion process in a 6 × 1 × 6 α-MoO_3_ supercell.

### Characterizations

Optical observations and Raman tests were conducted using a confocal microscope system (WITec, alpha 300 R). TEM and SAED images were obtained from a FEI Tecnai T20. HAADF-STEM images were acquired by a JEOL JEM-ARM300F TEM. XRD and XPS spectra were collected by a Bruker D8 Advance and a Nexsa surface analysis system (Thermo Scientific), respectively. Surface topography and near-field images were captured using a commercial s-SNOM (NeaSNOM, NeaSpec GmbH) setup with an atomic force microscope tip (NanoWorld, Arrow-NCPt) in tapping mode (~270 kHz frequency and ~70 nm amplitude). To eliminate edge-excited polaritons, flakes were rotated till the incident polarization was parallel to the edges.

## Supplementary information

Supplementary Information

## Data Availability

The data that support the findings of this study are available from the corresponding authors upon reasonable request.
