# Supplementary material for: Chemical switching of low-loss phonon polaritons in α-MoO3 by hydrogen intercalation
Source: Nat Commun. 2020 May 27;11:2646. doi: 10.1038/s41467-020-16459-3 (PMC7253429; doi:10.1038/s41467-020-16459-3)
Supplement: Supplementary file 1 — Supplementary Information [file 41467_2020_16459_MOESM1_ESM.pdf]

Supplementary Information

Chemical switching of low-loss phonon polaritons in α-MoO_3 by hydrogen intercalation

Wu et al.

Supplementary Information for

Chemical switching of low-loss phonon polaritons in α-MoO_3 by hydrogen intercalation

Yingjie Wu,[1,†] Qingdong Ou,[1,†] Yuefeng Yin,[1] Yun Li,[1] Weiliang Ma,[2] Wenzhi Yu,[1] Guanyu Liu,[3] * Xiaoqiang Cui,[4] Xiaozhi Bao,[5] Jiahua Duan,[6,7] Gonzalo Álvarez Pérez,[6,7] Zhigao Dai,[1] Babar Shabbir,[1] Nikhil Medhekar,[1] Xiangping Li,[3] * Chang-Ming Li,[8] Pablo Alonso-González,[6,7] Qiaoliang Bao[1]*

[1] Department of Materials Science and Engineering, and ARC Centre of Excellence in Future Low-Energy Electronics Technologies (FLEET), Monash University, Australia

[2] State Key Laboratory of Functional Materials for Informatics, Shanghai Institute of Microsystem and Information Technology, Chinese Academy of Sciences, China

[3] Guangdong Provincial Key Laboratory of Optical Fiber Sensing and Communications, Institute of Photonics Technology, Jinan University, China

[4] Laboratory of Automobile Materials of MOE, School of Materials Science and Engineering, Jilin University, China

[5] Joint Key Laboratory of the Ministry of Education, Institute of Applied Physics and Materials Engineering (IAPME), University of Macau, China

[6] Departamento de Física, Universidad de Oviedo, Spain

[7] Nanomaterials and Nanotechnology Center (CINN), Spain

[8] Institute of Advanced Cross-field Science, College of Life Science, Qingdao University, China

[†]These authors contributed equally to this work.

[*] Correspondence and requests for materials should be addressed to G. L. (email: liuguanyu@jnu.edu.cn) or to X. L. (email: xiangpingli@jnu.edu.cn) or to Q. B. (email: qiaoliang.bao@gmail.com).

1. Supplementary Notes

Supplementary Note 1. Linescan fitting and PhP lifetime extraction.

Before extracting the lifetimes of PhPs, we first consider the complex-valued optical signal $\boldsymbol{\sigma}$ after subtracting the background, as shown in Supplementary Eq. (1) which includes both tip- and edge-launched PhPs[1]:

$$\boldsymbol{\sigma}(x)=A\frac{e^{i2\mathbf{k}x}}{\sqrt{x}}+B\frac{e^{i\mathbf{k}x}}{x^{d}} \quad (1)$$

where $\mathbf{k}$ is the complex-valued wave vector, x is the distance from edge, d (~1) is variable decay, A and B are the parameters for tip- and edge-launched PhPs, respectively. Here, the factor $\sqrt{x}$ is used to compensate the circular-wave geometrical spreading of the tip-launched PhPs field while the factor x^d is for the edge-launched PhPs. We chose d from ref. 1 where a variable decay ~1 was investigated intensively for the edge-launched PhPs.

Therefore, to extract the lifetimes, Supplementary Eq. (1) can be converted into

$$\mathrm{Re}(\boldsymbol{\sigma}(x))=A\frac{e^{-2x\mathrm{Im}(\mathbf{k})}}{\sqrt{x}}\sin(2\mathrm{Re}(\mathbf{k})(x-x_c))+B\frac{e^{-x\mathrm{Im}(\mathbf{k})}}{x}\sin(\mathrm{Re}(\mathbf{k})(x-x_c^{'})) \quad (2)$$

where $\mathrm{Re}(\mathbf{k})$ and $\mathrm{Im}(\mathbf{k})$ are the real and imaginary parts of $\mathbf{k}$, x_c and $x_c^{'}$ are phase shifts, respectively. We notice that $\mathrm{Re}(\mathbf{k}) = 2\pi/\lambda_p$, where λ_p is the polaritosn wavelength, and propagation length $L = 1/\mathrm{Im}(\mathbf{k})$. Then, we obtain

$$\mathrm{Re}\big(\boldsymbol{\sigma}(x)\big)=A\frac{e^{\frac{-2x}{L}}}{\sqrt{x}}\sin\frac{4\pi(x-x_c)}{\lambda_p}+B\frac{e^{\frac{-x}{L}}}{x}\sin\frac{2\pi(x-x_c^{'})}{\lambda_p} \quad (3)$$

We thus use Supplementary Eq. (3) to fit the real part of third-harmonic near-field signals ($\mathrm{Re}(\boldsymbol{\sigma}_3)$) and extract λ_p and L for both L-RB and U-RB. Notably, the real part optical signal is extracted from both the amplitude and phase data according to $\boldsymbol{\sigma} = se^{i\varphi}$.[2] In the fitting procedure, a Levenberg Marquardt iteration algorithm is applied until a full convergence ($\Delta x^2 \leq 10^{-9}$) is achieved. Finally, the amplitude lifetime (τ) of PhPs can be obtained via $\tau = L/v_g$, where v_g ($= \partial\omega/\partial\mathrm{Re}(\mathbf{k})$) is group velocity extracted by taking the derivative from the polariton dispersion. Notably, for convenience, the amplitude signals are also used in the main text for the qualitative analysis and comparison of PhPs.

Supplementary Note 2. PhP evolution during the hydrogen intercalation.

From FFT results (Supplementary Fig. 9a, 900 cm^{-1}), it is clear that the periodicity of interference fringes gradually changes as increasing intercalation times. After 7-s

intercalation, no periodic interference fringes can be obtained both in s-SNOM images and FFT results, proving vanished PhPs in these flakes. In the 2-s and 5-s intercalation samples, parallel fringes can be observed (along the [100] direction), but the FWHM of the FFT peak is different from that of the original PhPs, indicating the changed PhP damping rate. After quantitative analysis in different frequencies (Supplementary Fig. 9b), it is demonstrated that PhP polariton wavelength and group velocitys in the 2-s flake have no obvious difference from those of the original flake. This result is consistent with the similar Raman shifts in these steps (Supplementary Fig. 10). However, PhP lifetimes and propagation lengths are shorter than those of original PhPs.

Numerical simulations are difficult to be conducted now, because of the unknown permittivity values of α-MoO_3 and H_xMoO_3. But we can make a qualitative description based on the Raman results. We attribute the chemical switching of PhPs to the perturbation of vibration modes (optical phonons). As shown in Supplementary Fig. 10, at the beginning (0 to 5 s) of hydrogenation, although the formation of the acicular H_xMoO_3 nanostructures can be observed in optical images, it has limited contribution to Raman vibrations. Infrared L-RB and U-RB of α-MoO_3 remain almost unchanged, so PhP dispersion has no obvious difference at this stage. However, the PhP scattering caused by acicular H_xMoO_3 leads to enhanced losses during PhP propagation. As the intercalation level increases (7 to 10 s), more H_xMoO_3 is formed, and the typical vibration peaks originating from H_xMoO_3 emerge in the Raman spectra, leading to the disappearance of PhP dispersion, although L-RB and U-RB can still be detected at this stage. To confirm our interpretation, we measured the Raman spectrum of a flake after 15-s hydrogenation, where the typical vibration peaks (L-BR and U-RB) of α-MoO_3 are fully undetectable (Supplementary Fig. 10). That is to say, there are no optical phonons or PhPs. Notably, considering a prolonged hydrogenation process could easily lead to irreversible CS (crystallographic shear) cracks, we precisely control the reaction time at 10 s in our reversible switching experiment.

Supplementary Note 3. Identification of the oriented acicular nanostructures.

Similar acicular "dark" structures in treated α-MoO_3 have been reported in several papers, but their composition has remained controversial. Smith[3] attributed these structures to <203> oriented type I H_xMoO_3 formed by the reaction of MoO_3 with alcohols. However, Liu[4] and Gai[5] identified these structures as planar oxygen vacancies, because of their hydrogen-free

reaction conditions. In our experiment, we prefer to identify these structures as type I H_xMoO_3. First, in the Raman spectra, the shifted vibration peaks, owing to the rearrangement of MoO_6 octahedra, are clearly observed and consistent with characteristic Raman peaks of type I H_xMoO_3. Second, in the XRD spectra, emerging peaks agree well with orthorhombic $H_{0.31}MoO_3$ (ICDD PDF: 70-0615). Third, in our spatially selective hydrogenation experiment (see Supplementary Fig. 18 and 19), no acicular structures can be observed in protected regions, indicating that these acicular structures are formed by the reaction of MoO_3 with hydrogen plasma. These evidences unambiguously demonstrate that, in our experiment, the obtained acicular structures are type I H_xMoO_3 nanostructures.

Then we turn attention to the formation of these oriented H_xMoO_3 nanostructures. We have already proved that these oriented needle-like nanostructures are caused by hydrogen intercalation. So we attribute the orientation to hydrogen diffusion in MoO_3. In our experiment, we observe that hydrogens diffuse along the <203> direction on the (010) plane, which makes it possible to interpret the three-dimension (3D) hydrogen diffusion pathways in type I H_xMoO_3. First, we analyze the hydrogen configurations in α-MoO_3 using density functional theory (DFT) calculations. At the low hydrogen concentration, neutron diffraction studies revealed that hydrogens are adsorbed on oxygens forming hydroxyl groups by weak hydrogen bonds.[6] So we calculate the adsorption energy (E_{ad}) by:

$$E_{ad}=E(MoO_3)+\frac{1}{2}E(H_2)\text{-}E(H/MoO_3) \quad (4)$$

Where $E(MoO_3)$, $E(H_2)$ and $E(H/MoO_3)$ are the total energies of bulk α-MoO_3, gas phase molecular hydrogen and H/α-MoO_3 system, respectively. We find that the O2 sites are the most energetically favourable (3.58 eV) for hydrogen adsorption in type I H_xMoO_3, as concluded in Supplementary Table 4.

In addition to the hydrogen diffusion pathway, the ^{1}H nuclear magnetic resonance (NMR) analysis provided the evidence that hydrogens diffuse along the zig-zag chains (along the [001] direction) of intralayer oxygen atoms within MoO_6 octahedra in the (100) plane.[7] It is also found that most hydrogens form the three-spin culsters[8] and locate alternatively within the zig-zag chains because of the cationic repulsion[9]. For calculational convenience, we investigate the 3D hydrogen diffusion pathway by calculating the thermodynamic stability of two hydrogen atoms. As shown in Supplementary Fig. 13, we compare the stabilities of two configurations: the literature reported [001] direction and our observed [203] direction. We

find that the [203] direction is slightly energetically favorable (15 meV) than the [001] direction, possibly caused by the cationic repulsion. Notably, in our experiment, hydrogens are inserted into MoO_6 octahedra (intralayers), which is different from the metal-atom intercalated α-MoO_3, where intercalation mainly happens within vdW gaps (interlayers)[10].

Supplementary Note 4. PhP behaviours near the acicular nanostructures.

The intercalated MoO_3 flake with acicular H_xMoO_3 nanostructures is an ideal platform to investigate the PhP behaviour at in-plane domain boundaries. As shown in Supplementary Fig. 16a, we analyse two typical domains in L-RB: the edge-H_xMoO_3 (EH) domain and the H_xMoO_3-H_xMoO_3 (HH) domain, which are indicated by the green and red rectangles, respectively. In the EH domain, two different PhPs can be observed: one is along the [100] direction (black bold arrow) and the other is along the [302] direction (cyan bold arrow). However, in the HH domain, only the [302] propagated PhPs can be observed (pink bold arrow). The obtained PhPs are coupled by a variety of polaritons launched or reflected by tips, edges and H_xMoO_3 nanostructures. Notably, the reflection of hyperbolic PhPs in our experiment is quite interesting but complicated because it is not a simple mirror reflection. The hyperbolic reflection mechanism may be studied in our future work. Interestingly, we find that these needle-like nanostructures can not only reflect but also launch PhPs, according to the both tip and edge launched PhPs observed in the extracted linescan, as shown in Supplementary Fig. 16c.

Now we turn our attention to refraction. As shown in Supplementary Fig. 16b, in the central region of flakes (white rectangle), the PhPs in the original α-MoO_3 slab is difficult to be observed, because of the unavoidable loss during propagation. By contrast, in the intercalated slab, PhPs can be clearly observed, proving that the PhPs perpendicular to the acicular H_xMoO_3 is not induced by the refraction of the [100] oriented PhPs. In addition to U-RB, the obtained amplitude image (Supplementary Fig. 17) is similar to that in L-RB, but the PhPs at boundaries follow a different mechanism, as the elliptical propagation in U-RB, in which PhPs along the [001] direction should also be considered.

Supplementary Note 5. Novelty of this work compared with Sn-MoO_3.

Sn intercalation has been used to manipulate PhPs in α-MoO_3,[10] but we would like to emphasie the novelty of our work through two main differences with the previous Sn-MoO_3

work. First we use hydrogen as the intercalator and our intercalation strategy is a vapour-phase process. Our solvent-free intercalation system can effectively avoid damage and contaminants as well as keep the MoO_3 flakes clean, which is beneficial to the following precise s-SNOM measurement. More importantly, by our hydrogen intercalation method, we can manipulate PhPs in MoO_3 through three different strategies: reversible switching of PhPs, construction of the in-plane nano-antennas and nano-cavities, and spatially controlled switching of PhPs.

2. Supplementary Figures

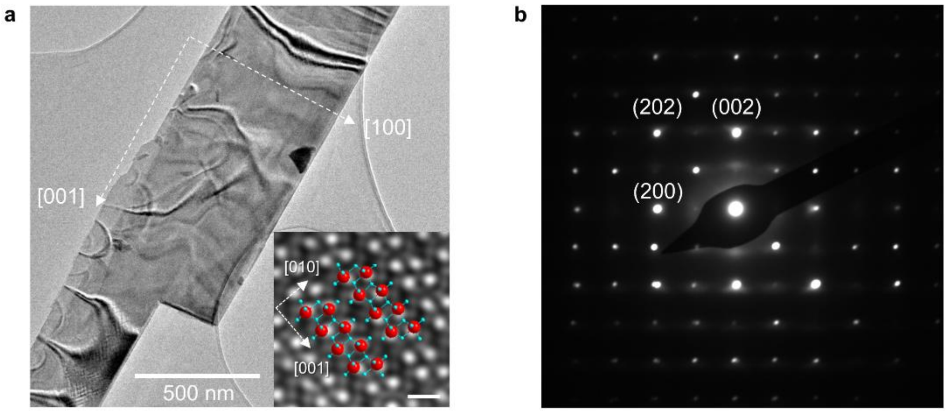

Supplementary Figure 1. a, TEM and HAADF STEM (inset, scale bar, 5 Å) images of prepared α-MoO_3 slabs. The bright dots in the HAADF-STEM image are Mo atoms, whose alignment is in good agreement with the ball-and-stick model (red ball represents Mo) of α-MoO_3. **b**, SAED pattern of the obtained α-MoO_3 slabs, indicating the orthorhombic crystal structure and good crystallinity.

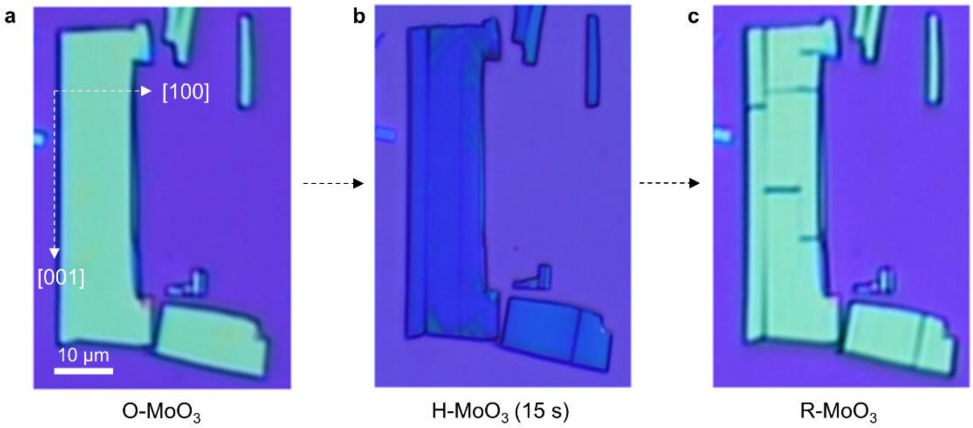

Supplementary Figure 2. Microphotographs of MoO_3 slabs before hydrogenation (**a**), after 15-s hydrogenation (**b**) and after dehydrogenation (**c**), respectively. It is clear that straight cracks oriented along the [001] direction appeared on the slab after hydrogenation. These cracks are caused by the crystallographic shear (CS) planes.[3] After dehydrogenation, CS cracks cannot be repaired. Besides, new cracks along the [100] direction emerge, caused by the rearrangement of lattices and inner stress. To avoid imposing unrecoverable damage to flakes, one should control the intercalation parameters carefully.

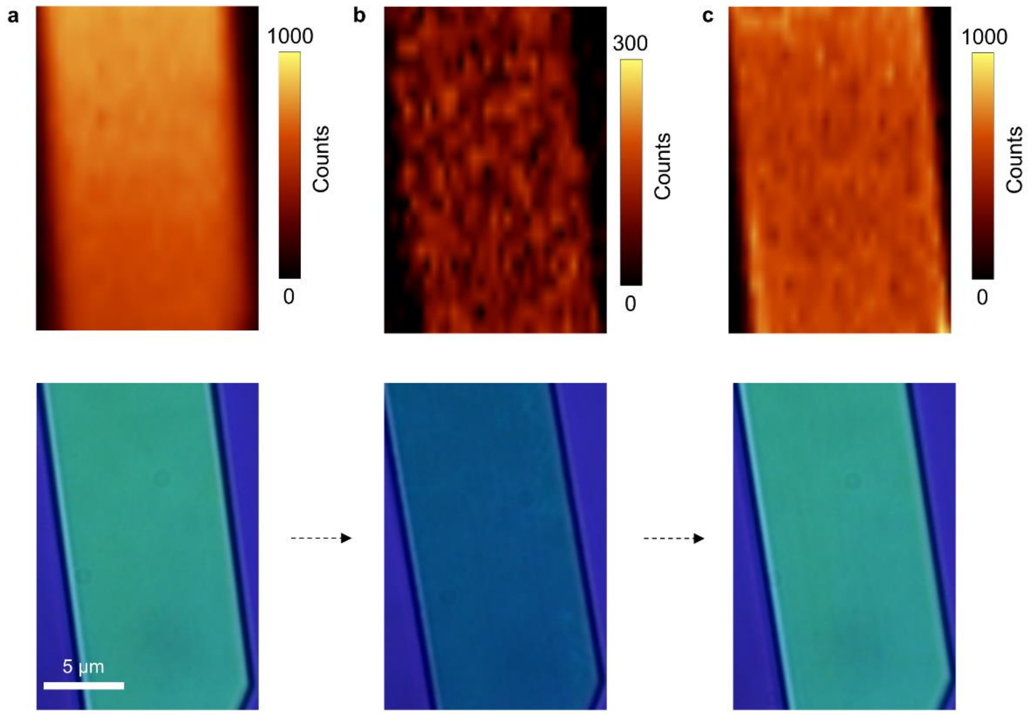

Supplementary Figure 3. Raman intensity mappings (top) and corresponding microphotographs (bottom) of a MoO_3 flake before intercalation (**a**), after 10-s intercalation (**b**) and after deintercalation (**c**), respectively. Raman intensity mappings were obtained from 795 to 840 cm^{-1} (Mo-O2' vibration peak, L-RB) at the same laser power. It is clear that after intercalation, the intensity of Mo-O2' vibration peak is weakened throughout the whole flake, which can be enhanced after deintercalation. Non-uniform distribution of intensity was observed in the mapping of R-MoO_3, which was possibly caused by the unavoidable defects during thermal treatment.

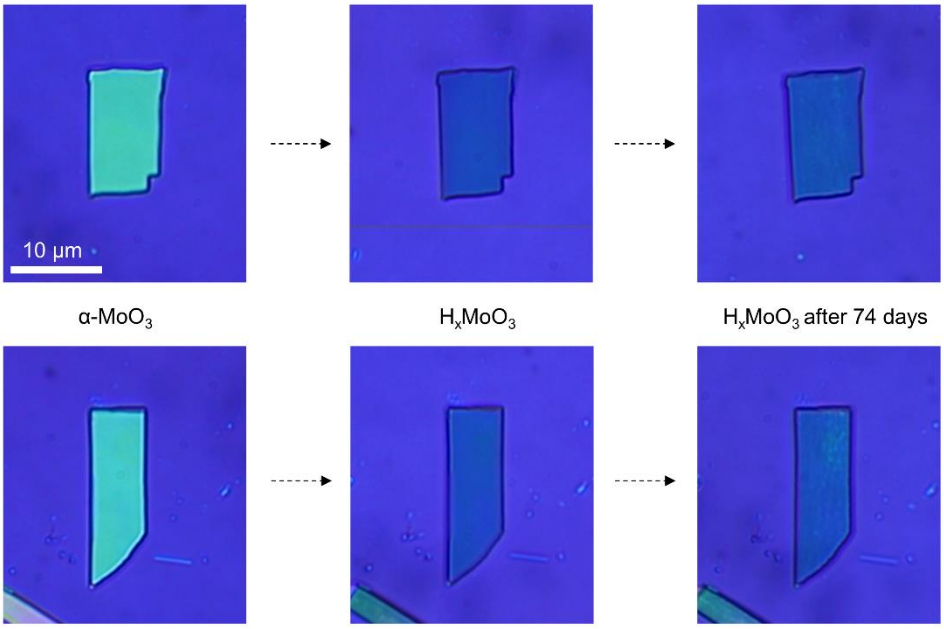

Supplementary Figure 4. Optical images of the hydrogen intercalated (10 s) MoO_3 flakes after 74 days in the ambient atmosphere.

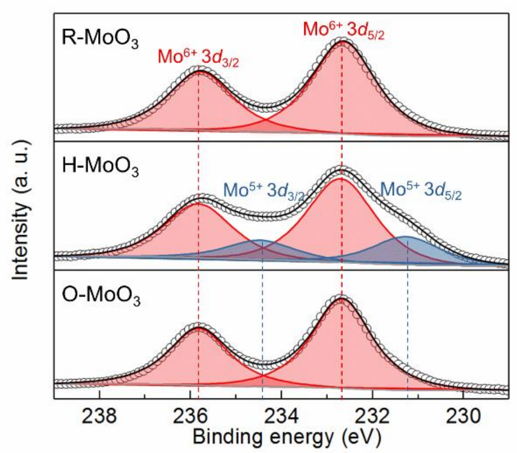

Supplementary Figure 5. XPS analysis of O-MoO_3, H-MoO_3 (10 s) and R-MoO_3. In H-MoO_3, Mo^{6+} is partially reduced to Mo^{5+} by hydrogen insertion. The calculated proportion of Mo^{5+} is 25%. After dehydrogenation, Mo^{5+} is oxidized to Mo^{6+}.

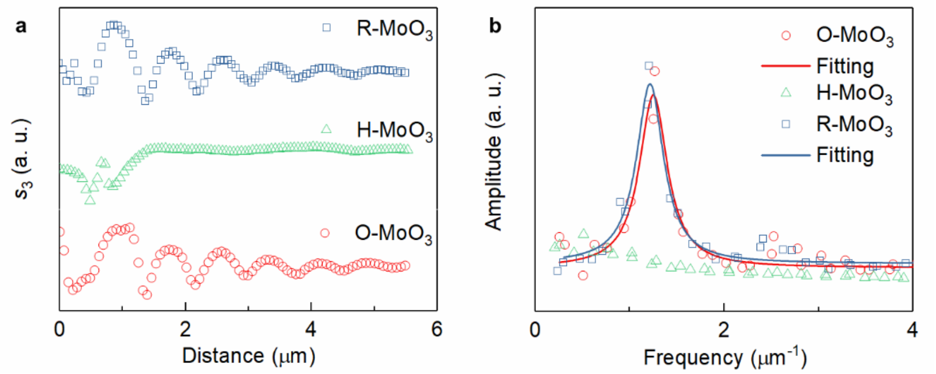

Supplementary Figure 6. **a**, Line-scan amplitude curves (black dashed lines in Fig. 2b) of the original, hydrogenated and recovered MoO_3. **b**, Fast Fourier transform (FFT) and Lorentzian fitting results of extracted line scans. The raw FFT data in original and recovered flakes exhibit a sharp peak at 1.25 ± 0.02 and 1.21 ± 0.02 μm^{-1}, respectively, indicating the predominant tip-launched PhPs in scanned amplitude images.[11] Besides, Lorentzian fitting curves with similar FWHM values (0.36 ± 0.03 and 0.35 ± 0.03 μm^{-1}) prove the consistent PhP propagation in the original and recovered MoO_3.[12] By contrast, no obvious peaks can be observed in the H-MoO_3 FFT, clearly demonstrating that the PhP feature has been switched after hydrogen intercalation.

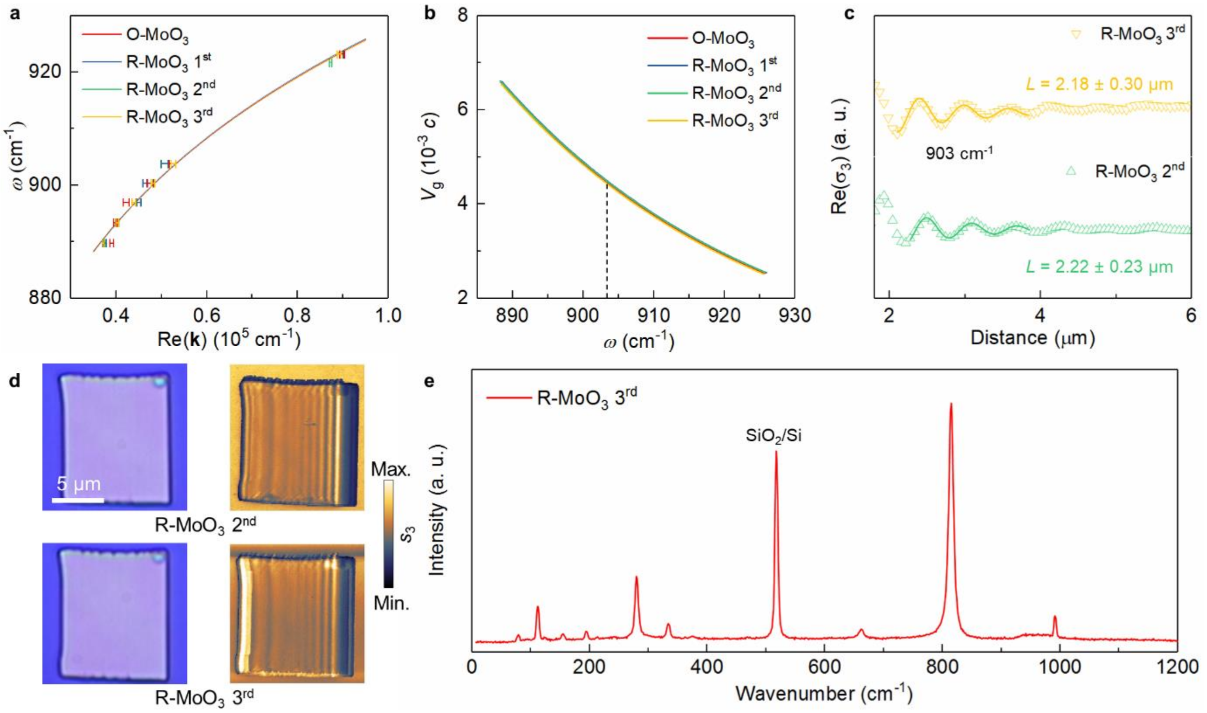

Supplementary Figure 7. Quantitative analysis of the dispersion data (**a**), group velocities (**b**) and propagation lengths (**c**) of PhPs in L-RB along the [100] direction obtained from the 2nd and 3rd R-MoO_3 flakes, respectively. The error bars define the 95% confidence intervals. **d**, Microphotographs and amplitude images (890 cm^{-1}) of the 2nd and 3rd R-MoO_3 flakes. **e**, Raman spectrum of the 3rd R-MoO_3 flake, exhibiting characteristic vibration features of α-MoO_3. The shift around 520 cm^{-1} is assigned to the SiO_2/Si substrate.[13]

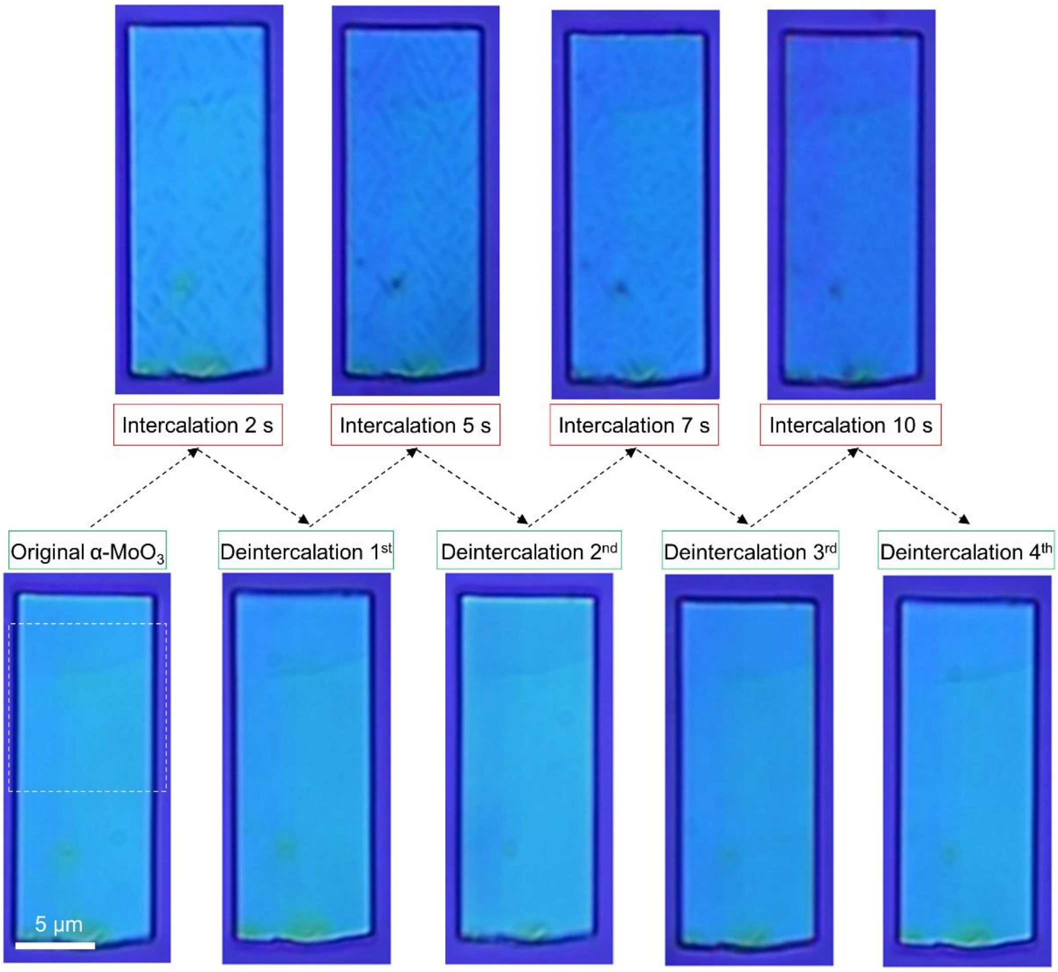

Supplementary Figure 8. Illustration of experiment processes and corresponding microphotographs in each step. The white rectangle indicates the region displayed in Fig. 4b. The morphologies and distributions of acicular H_xMoO_3 nanostructures and the contrasts of flakes slowly change with increasing intercalation times. However, the contrasts and geometries of flakes after each deintercalation process remain almost unchanged.

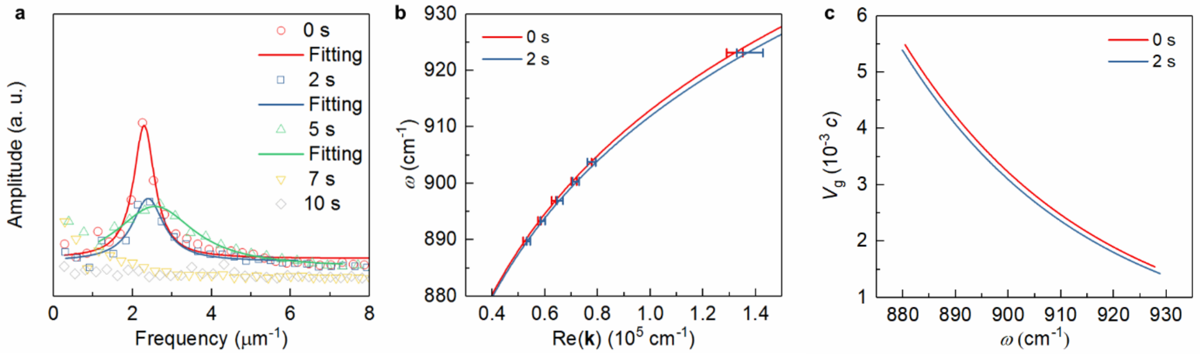

Supplementary Figure 9. **a**, FFT and Lorentzian fitting results of the line traces shown in Fig. 4c. **b**, PhP dispersion data from the original and hydrogenated (2 s, [100] direction) flakes. The error bars represent the 95% confidence intervals. **c**, Calculated group velocities. The PhP group velocities (900 cm^{-1}) in 0- and 2-s flakes are 3.22×10^{-3} *c* and 3.09×10^{-3} *c*, respectively.

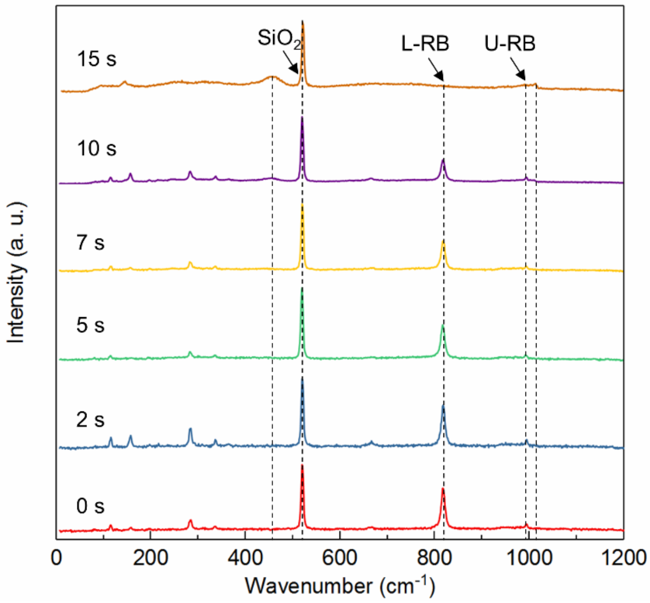

Supplementary Figure 10. Raman spectra (normalized to SiO_2 substrate) of the original and intermediate MoO_3 flakes during hydrogenation.

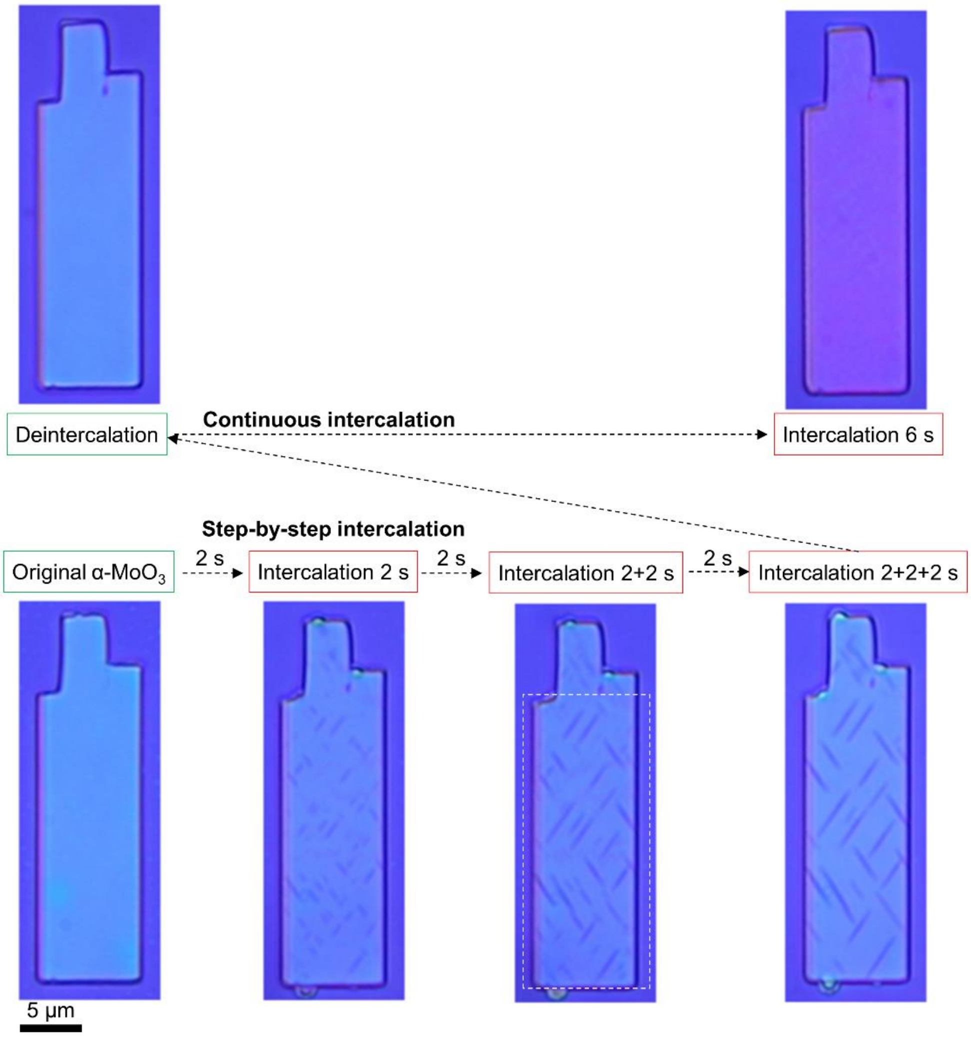

Supplementary Figure 11. Comparison between the continuous intercalation and step-by-step intercalation approaches. White rectangle indicates the region displayed in Fig. 4d. During the step-by-step intercalation process, the scales (especially lengths) of needle-like nanostructures gradually elongate with increasing time. In contrast, continuous intercalation (under the same reaction time) leads to relatively uniform hydrogen distribution.

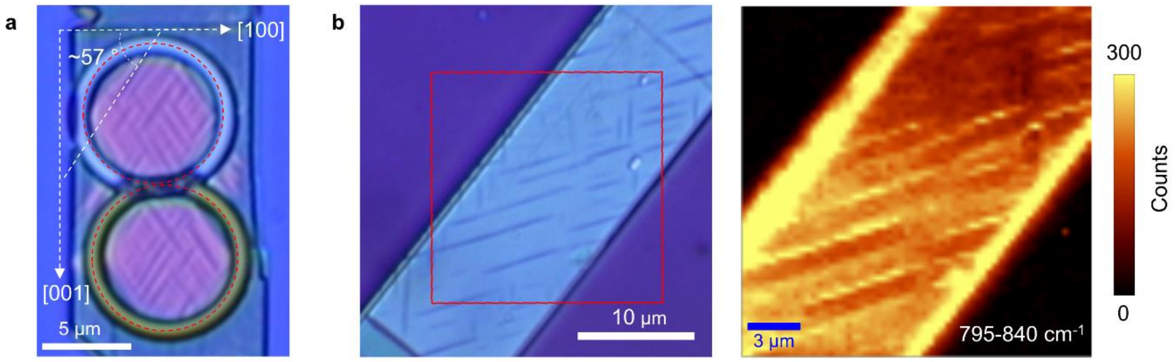

Supplementary Figure 12. **a**, Microphotograph of H-MoO_3 discs (red dashed cycles). The MoO_3 discs are fabricated by focused ion beam (FIB) lithography technology. The orientation of needle-like fringes is along 57 °respect to the [100] direction, proving that the hydrogen intercalation direction is dependent on the crystal structure of α-MoO_3 instead of the morphologies of slabs. **b**, Microphotograph and corresponding Raman intensity mapping (795-840 cm^{-1}, Mo-O2' vibration mode) of a 5-s intercalation flake. In the Raman mapping, the relative intensity of acicular H_xMoO_3 nanostructures is weaker than the intercalation-free regions, indicating the disturbance of the Mo-O2' vibration mode in H-MoO_3.[4]

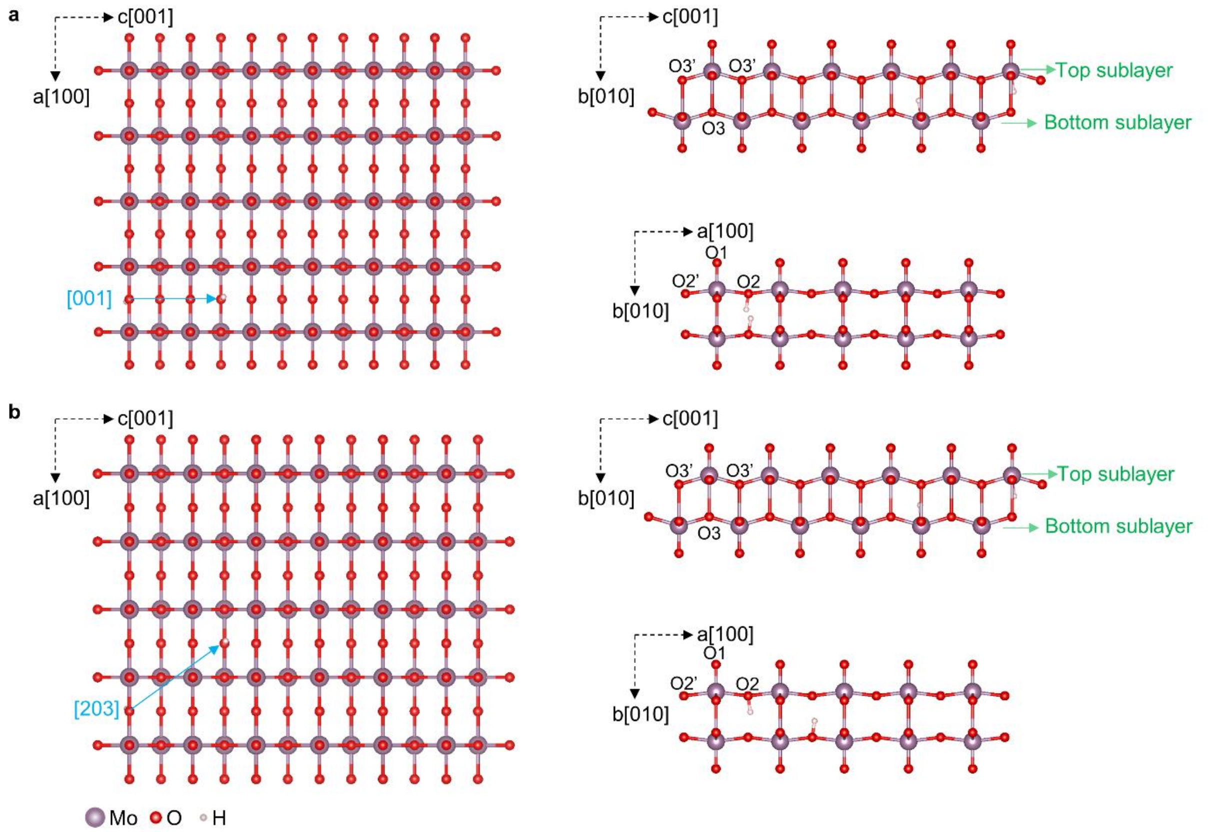

Supplementary Figure 13. Schematics of the intralayer hydrogen diffusion pathways along the [001] (**a**) and [203] (**b**) directions, respectively.

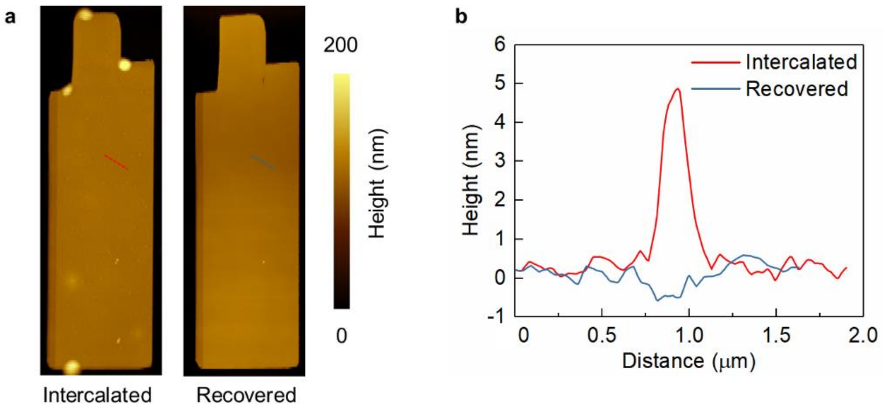

Supplementary Figure 14. a, Height topographies of the intercalated (2+2 s) and recovered MoO_3 flakes. **b**, Thickness profiles obtained from the dashed lines in **a**. These results indicate that the needle-like nanostructures can also be recovered via our reversible hydrogenation method.

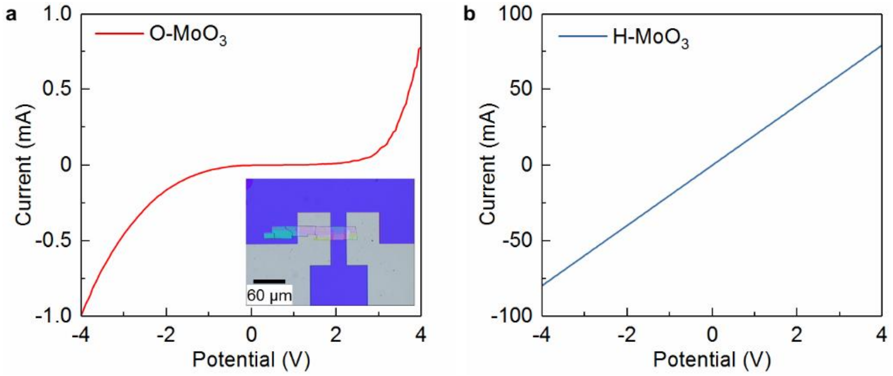

Supplementary Figure 15. I-V curves of an α-MoO_3 slab before (**a**) and after (**b**) hydrogen intercalation, respectively. Inset, the device optical image.

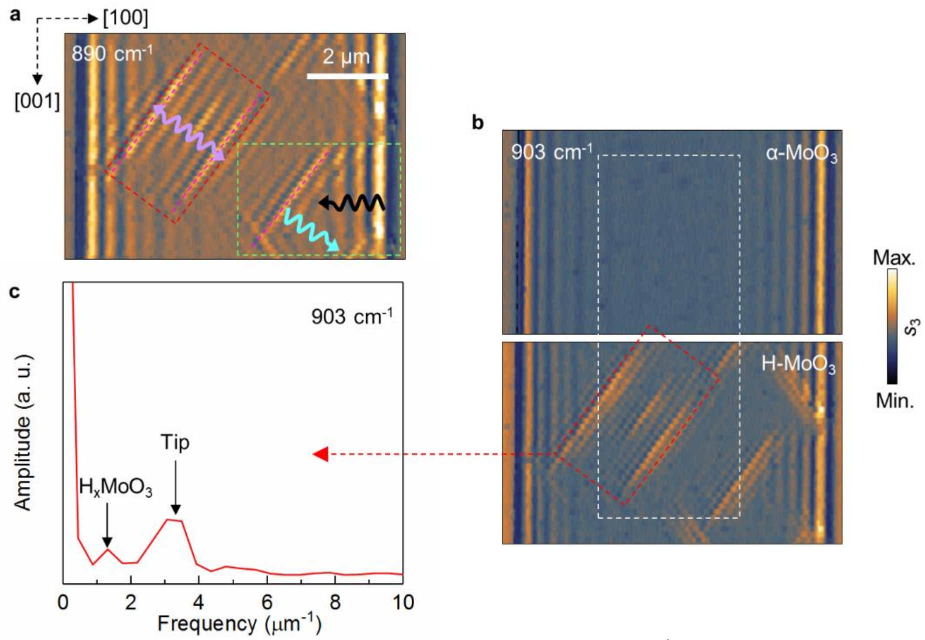

Supplementary Figure 16. **a**, Amplitude image at 890 cm^{-1}. **b**, Amplitude images of the same region in the original and intercalated (2+2 s) MoO_3 flakes at 903 cm^{-1}. **c**, FFT result of the line profile extracted from the red rectanglar region (903 cm^{-1}) in **b**.

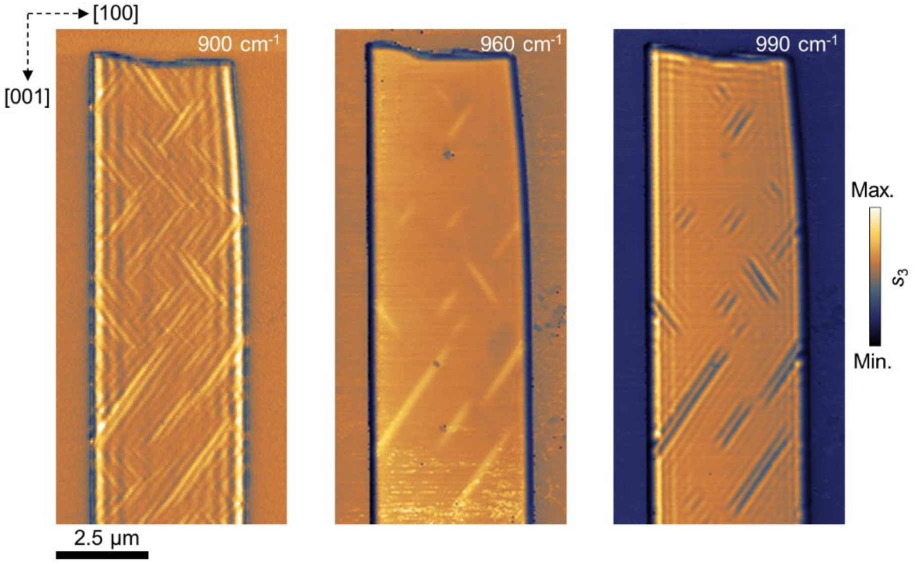

Supplementary Figure 17. Amplitude images of an intercalated (2+2 s) MoO_3 flake (~120 nm) at different frequencies. In the amplitude image at 960 cm^{-1}, the polaritonic fringes are almost invisible, while the needle-like nanostructures have stronger contrast than the hydrogen-free regions, indicating the increased metallicity.

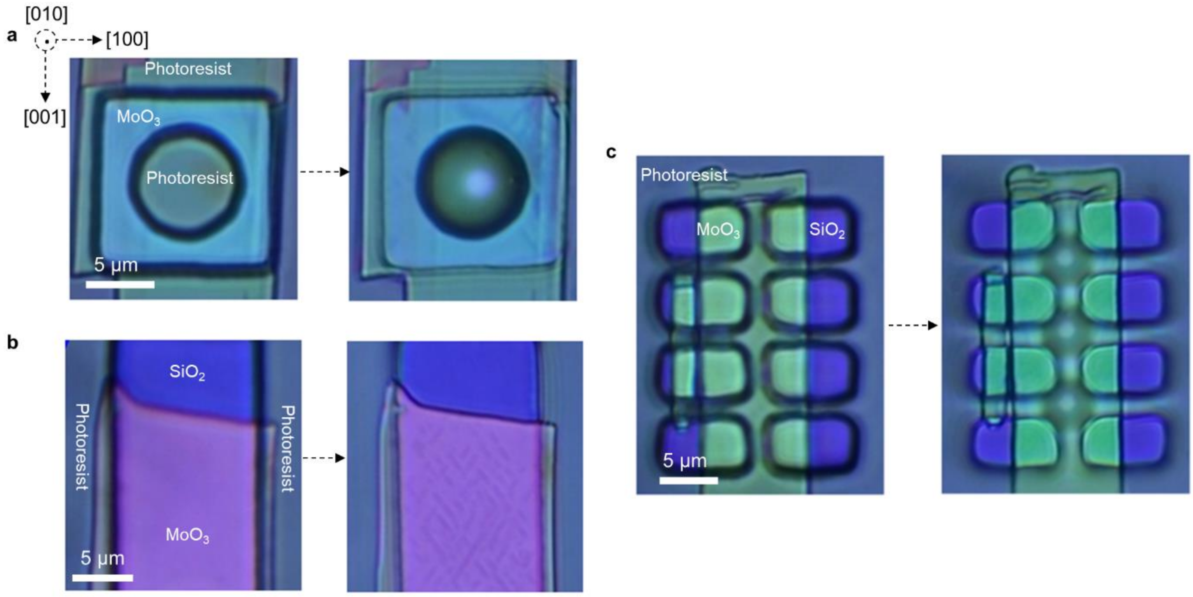

Supplementary Figure 18. Comparison of hydrogen intercalation in α-MoO_3 slabs along different directions. Left, slabs before intercalation; right, slabs after 10-s intercalation. Controlled intercalation is realised by covering different edges (planes) using photoresist with the same thickness (~1 μm). It is clear that, the optical contrast of the slab with (010) plane exposed has no obvious difference after intercalation. However, in the flake with (010) and (001) planes exposed, acicular nanostructures merge after intercalation. In Supplementary Fig. 18c, the contrast of exposed regions ((010) and (100) planes) becomes waker uniformly after intercalation, which is similar to the previously mentioned slabs without any protection. We can thus have a qualitative conclusion that the hydrogen intercalation rate (v) in our experiment follows $v_{(100)} > v_{(001)} > v_{(010)}$, which are mainly determined by the crystal structure rather than the surface area. Further systematic and controlled experiments are needed to have a more comprehensive understanding on this.

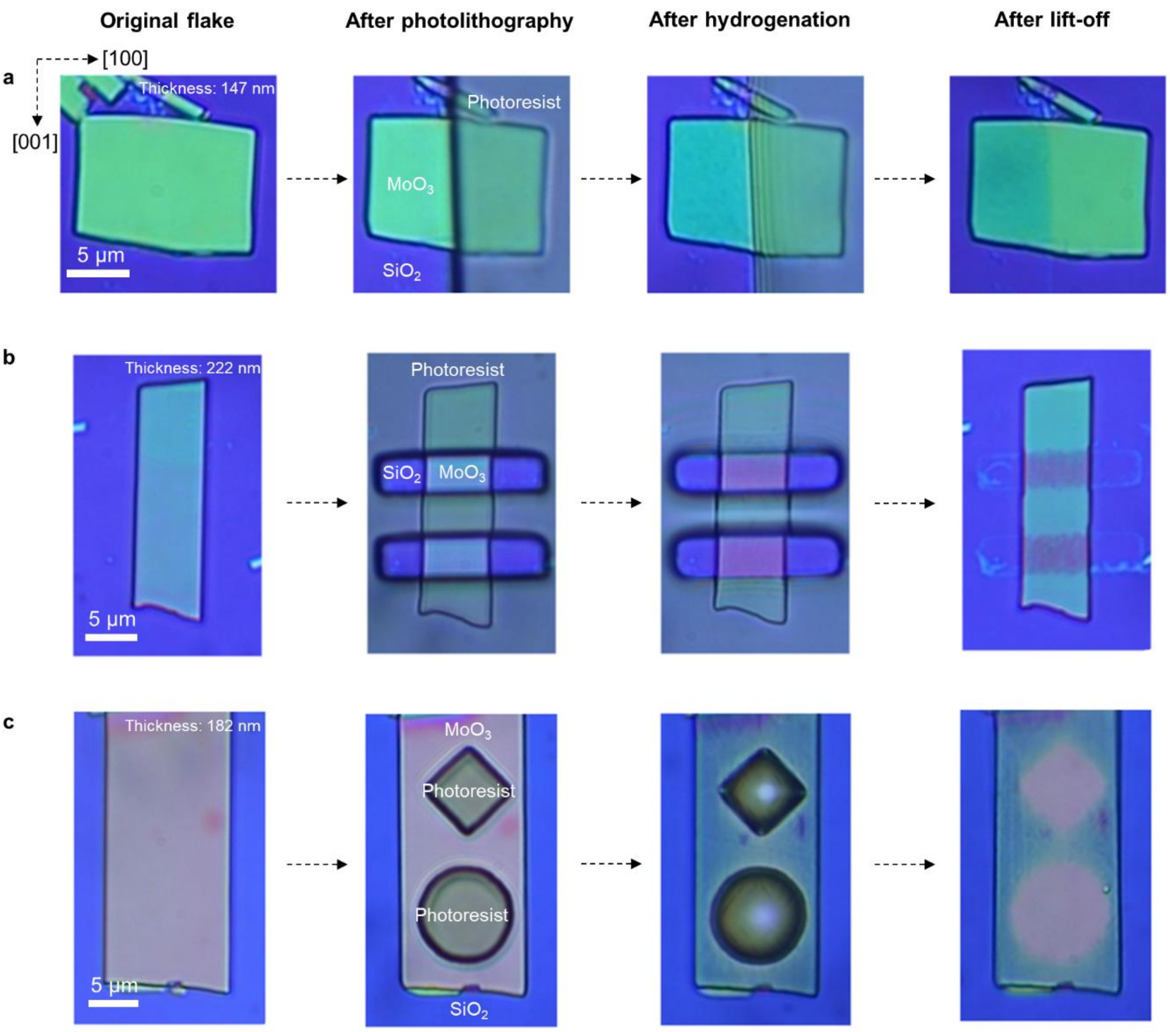

Supplementary Figure 19. Microphotographs of the flakes at each step during the spatially controlled intercalation process. **a**, In-plane heterostructure. The left part is exposed to and the right part is protected by photoresist. **b**, Array. The pink-color regions in the right two figures are hydrogenated MoO_3. **c**, Disc- and square-shape photoresists are placed on the α-MoO_3 flake so as to achieve desired patterns. The light pink-color regions in the right figure are intrinsic α-MoO_3 without intercalation (or with minimum effect from intercalation).

3. Supplementary Tables

Supplementary Table 1. Binding energies and full width at half maximum (FWHM) values of Mo in the different samples from XPS curves.

Samples	Mo^{6+}			Mo^{5+}			Mo^{5+} ratio
	$3d_{3/2}$ (eV)	$3d_{5/2}$ (eV)	FWHM (eV)	$3d_{3/2}$ (eV)	$3d_{5/2}$ (eV)	FWHM (eV)	
**O-MoO$_3$**	235.88	232.74	1.61	-	-	-	0
**H-MoO$_3$**	235.80	232.78	1.82	234.45	231.23	1.82	25%
**R-MoO$_3$**	235.85	232.70	1.72	-	-	-	0

Supplementary Table 2. Fitting parameters for the PhP dispersion fittings in Fig. 2c and Fig. 3b in the main text.

Samples	L-RB		U-RB			
	[100]		[100]		[001]	
	a	*b*	*a*	*b*	*a*	*b*
**O-MoO$_3$**	927.706 ±1.772	0.041 ±0.003	996.118 ±0.189	0.010 ±0.004	994.957 ±0.189	0.011 ±0.005
**R-MoO$_3$ 1st**	927.922 ±1.772	0.042 ±0.002	996.435 ±0.373	0.010 ±0.001	995.275 ±0.188	0.011 ±0.004
**R-MoO$_3$ 2nd**	927.785 ±0.483	0.042 ±0.001	-	-	-	-
**R-MoO$_3$ 3rd**	927.493 ±1.326	0.041 ±0.002	-	-	-	-

Supplementary Table 3. Obtained binding energies and FWHM values of Mo in samples with different intercalation times. XPS curves are displayed in Fig. 4a in the main text.

Samples	Mo^{6+}			Mo^{5+}			Mo^{5+} ratio
	$3d_{3/2}$ (eV)	$3d_{5/2}$ (eV)	FWHM (eV)	$3d_{3/2}$ (eV)	$3d_{5/2}$ (eV)	FWHM (eV)	
**O-MoO$_3$**	235.88	232.74	1.61	-	-	-	0
**H-MoO$_3$ 2s**	235.83	232.70	1.50	234.54	231.21	1.50	15%
**H-MoO$_3$ 5s**	235.82	232.69	1.57	234.46	231.11	1.57	17%
**H-MoO$_3$ 7s**	235.75	232.68	1.69	234.46	231.09	1.69	18%
**H-MoO$_3$ 10s**	235.80	232.78	1.82	234.45	231.23	1.82	25%

Supplementary Table 4. Hydrogen adsorption energies on the O1, O2 and O3 sites.

Configurations	Adsorption energy (eV)
O1	3.06
O2	3.58
O3	1.13

Supplementary Table 5. Comparison of different PhPs tuning methods with our approach.

**Methods**	**Mechanisms and geometries**	**PhPs**	**Tunabilities**	**Refs.**
**Isotopically pure h-BN**	• Vibration & permittivity variations<br>• Different slabs	• Hyperbolic PhPs<br>• Low loss	• Prolonged lifetimes (ps)<br>• Discrete tuning (isotopic content)	12
**Suspended h-BN**	• Dielectric environment<br>• Suspended structure	• Hyperbolic PhPs<br>• Low loss	• Elongated polariton wavelength<br>• Reduced damping	14
**h-BN/BP**	• Dielectric environment<br>• Vertical heterostructure	• In-plane anisotropy	• Convert in-plane isotropic PhPs to in-plane anisotropic PhPs	15
**Sn-MoO_3**	• Metal atoms scattering (chemical)<br>• Doped structure (uncontrollable)	• Low loss<br>• Long lifetime (ps)<br>• In-plane anisotropy	• Reduced lifetimes<br>• Decreased polariton wavelengths	10
**h-BN/VO_2**	• Dielectric environment (thermal)<br>• Vertical heterostructure	• Hyperbolic PhPs	• Reconfigurable tuning (thermal cycling)<br>• Continuous tuning (temperature)	16,17
**GST/Quartz**	• Dielectric environment (optical)<br>• Vertical heterostructure	• Ultra-confined PhPs	• Reversible switching (laser pulse)<br>• Scalable switching	18
**h-BN/GST**	• Dielectric environment (optical)<br>• Vertical heterostructure	• Hyperbolic PhPs<br>• Low loss	• Polariton wavefront engineering<br>• Rewritable waveguides	19
**H-MoO_3**	• Lattice vibrations (chemical)<br>• Within an individual slab<br>• In-plane heterostructure	• Low loss<br>• Long lifetime (ps)<br>• In-plane anisotropy	• Reversible switching<br>• Spatially controllable switching<br>• Continous tuning (intercalation time)<br>• *In situ* switching (spillover)	This work

4. Supplementary References

1. Woessner, A. et al. Highly confined low-loss plasmons in graphene-boron nitride heterostructures. *Nat. Mater.* **14**, 421-425 (2015).
2. Hillenbrand R. and Keilmann F. Complex optical constants on a subwavelength scale. *Phys. Rev. Lett.*, **85**, 3029-3032 (2000).
3. Smith, R. L. & Rohrer, G. S. The protonation of MoO_3 during the partial oxidation of alcohols. *J. Catal.* **173**, 219-228 (1998).
4. Liu, H., Lee, C. J. J., Guo, S. & Chi, D. New insights into planar defects in layered alpha-MoO_3 crystals. *Langmuir* **34**, 14003-14011 (2018).
5. Gai, P. L. Dynamic studies of metal oxide catalysts: MoO_3. *Philos. Mag. A* **43**, 841-855 (2006).
6. Dickens, P. G., Birtill, J. J. & Wright, C. J. Elastic and inelastic neutron studies of hydrogen molybdenum bronzes. *J. Solid State Chem.* **28**, 185-193 (1979).
7. Ritter, C., Müller-Warmuth, W., Spiess, H. W. & Schöllhorn, R. Quasi-one-dimensional behaviour of hydrogen in $H_{0.35}MoO_3$ and $H_{0.33}WO_3$ as revealed by proton NMR. *Ber. Bunsenges. Phys. Chem.* **86**, 1101-1106 (1982).
8. Kunitomo, M., Eda, K., Sotani, N. & Kaburagi, M. Protonic location in hydrogen molybdenum bronzes H_xMoO_3 as studied by proton NMR lineshape analysis. *J. Solid State Chem.* **99**, 395-403 (1992).
9. Zeng, H. C., Xie, F., Wong, K. C. & Mitchell, K. A. R. Insertion and removal of protons in single-crystal orthorhombic molybdenum trioxide under H_2S/H_2 and O_2/N_2. *Chem. Mater.* **14**, 1788-1796 (2002).
10. Zheng, Z. et al. Highly confined and tunable hyperbolic phonon polaritons in van der Waals semiconducting transition metal oxides. *Adv. Mater.* **30**, 1705318 (2018).
11. Ma, W. et al. In-plane anisotropic and ultra-low-loss polaritons in a natural van der Waals crystal. *Nature* **562**, 557-562 (2018).
12. Giles, A. J. et al. Ultralow-loss polaritons in isotopically pure boron nitride. *Nat. Mater.* **17**, 134-139 (2018).
13. Nikitin, T. and Khriachtchev, L. Optical and structural properties of Si nanocrystals in SiO_2 films. *Nanomaterials*, **5**, 614-655 (2015).
14. Dai, S. et al. Hyperbolic phonon polaritons in suspended hexagonal boron nitride. *Nano Lett.* **19**, 1009-1014 (2019).
15. Chaudhary, K. et al. Engineering phonon polaritons in van der Waals heterostructures to enhance in-plane optical anisotropy. *Sci. Adv.* **5**, eaau7171 (2019).

16. Folland, T. G. et al. Reconfigurable infrared hyperbolic metasurfaces using phase change materials. *Nat. Commun.* **9**, 4371 (2018).
17. Dai, S. et al. Phase-change hyperbolic heterostructures for nanopolaritonics: A case study of hBN/VO_2. *Adv. Mater.* **31**, 1900251 (2019).
18. Li, P. et al. Reversible optical switching of highly confined phonon-polaritons with an ultrathin phase-change material. *Nat. Mater.* **15**, 870-875 (2016).
19. Chaudhary, K. et al. Polariton nanophotonics using phase change materials. *Nat. Commun.* **10**, 4887 (2019).
